# Bee Products: A Representation of Biodiversity, Sustainability, and Health

**DOI:** 10.3390/life11090970

**Published:** 2021-09-15

**Authors:** Alessandra Durazzo, Massimo Lucarini, Manuela Plutino, Luigi Lucini, Rita Aromolo, Erika Martinelli, Eliana B. Souto, Antonello Santini, Giuseppe Pignatti

**Affiliations:** 1 CREA-Research Centre for Food and Nutrition, via Ardeatina 546, 00178 Rome, Italy; massimo.lucarini@crea.gov.it; 2 CREA-Research Centre for Forestry and Wood, Viale Santa Margherita 80, 52100 Arezzo, Italy; manuela.plutino@crea.gov.it; 3 Department for Sustainable Food Process, Università Cattolica del Sacro Cuore, via Emilia Parmense 84, 29122 Piacenza, Italy; luigi.lucini@unicatt.it (L.L.); erika.martinelli1@unicatt.it (E.M.); 4 CREA-Research Centre for Agriculture and Environment, via Della Navicella 4, 00184 Rome, Italy; rita.aromolo@crea.gov.it; 5 Department of Pharmaceutical Technology, Faculty of Pharmacy, University of Coimbra, Pólo das Ciências da Saúde, Azinhaga de Santa Comba, 3000-548 Coimbra, Portugal; souto.eliana@gmail.com; 6 CEB-Centre of Biological Engineering, Campus de Gualtar, University of Minho, 4710-057 Braga, Portugal; 7 Department of Pharmacy, University of Napoli Federico II, via D. Montesano 49, 80131 Napoli, Italy; asantini@unina.it; 8 CREA-Research Centre for Forestry and Wood, via Valle Della Quistione 27, 00166 Rome, Italy; giuseppe.pignatti@crea.gov.it

**Keywords:** bee products, honey, biodiversity, sustainability, ecosystem services, health, bioindicators

## Abstract

Biodiversity strengthens the productivity of any ecosystem (agricultural land, forest, lake, etc.). The loss of biodiversity contributes to food and energy insecurity; increases vulnerability to natural disasters, such as floods or tropical storms; and decreases the quality of both life and health. Wild and managed bees play a key role in maintaining the biodiversity and in the recovery and restoration of degraded habitats. The novelty character of this perspective is to give an updated representation of bee products’ biodiversity, sustainability, and health relationship. The role of bees as bioindicators, their importance in the conservation of biodiversity, their ecosystem services, and the variety of the bee products are described herein. An overview of the main components of bee products, their biological potentials, and health is highlighted and detailed as follows: (i) nutritional value of bee products, (ii) bioactive profile of bee products and the related beneficial properties; (iii) focus on honey and health through a literature quantitative analysis, and (iv) bee products explored through databases. Moreover, as an example of the interconnection between health, biodiversity, and sustainability, a case study, namely the “Cellulose Park”, realized in Rome (Italy), is presented here. This case study highlights how bee activities can be used to assess and track changes in the quality of agricultural ecosystems—hive products could be valid indicators of the quality and health of the surrounding environment, as well as the changes induced by the biotic and abiotic factors that impact the sustainability of agricultural production and biodiversity conservation in peri-urban areas.

## 1. Introduction

A close correlation exists between the potential benefits, the “functional role” of food, and the territory. The linkage between nutrition and the environment through biodiversity and sustainability has been investigated. Nutrition, health, and environmental sustainability are strictly linked throughout the food system. Assessments of the environmental protection such as soil quality and landscaping, ecological resources, animal wellness, and appropriate farming-based land-use, have led to an improvement of the product quality [1,2]. This perspective aims at giving an updated look into bee products, biodiversity, sustainability, and health relationships. The role of bees as bioindicators in the conservation of biodiversity, recovery, and the restoration of degraded habitats and supply ecosystem services is known. In this respect, a delineation of the variety of bee products in a range of floral resources and diversity of territories is commented. The nutritional value, bioactive components, and beneficial properties of bee products are described. Finally, an example of the interconnection of health, biodiversity, and sustainability is given as a case study using the “Cellulose Park“ realized in Rome (Italy). This case study highlights how bees’ activities can be used to assess and track changes in the quality of agricultural ecosystems, and could represent a valid indicator of the quality and health of the surrounding environment as well as the changes induced by biotic and abiotic factors. The importance of research in this area has been documented by a comprehensive literature search analysis outlining the interconnection between health, biodiversity, and sustainability.

## 2. Linkage between Bees, Ecosystems, and Biodiversity: A Snapshot

### 2.1. Role of Bees as Bioindicators and Conservation of Biodiversity

Biodiversity is defined as “the variety of life on Earth and the natural patterns it forms” [3]. According to Norse et al. [4], biodiversity can be subdivided into three separate levels: ecosystem, specific, and genetic.

Assessment of the biodiversity is generally made through indicators that are defined as bioindicators in the specific case. Many pollinators are bioindicators. Bees also belong to this category. According to some authors [5,6,7], a bioindicator should have the characteristics specified in Table 1.

Other authors [9] have highlighted that, if taken individually, bioindicators tell us little about the overall environmental trends.

Landres et al. [10] recommended the following: (i) to use indicators as part of a broader strategy of analysis on the key habitats (corridors or other landscape structures) and species, and (ii) to include monitoring indicators of structural, compositional, and functional biodiversity at multiple organization levels.

However, biodiversity strengthens the productivity of any ecosystem (agricultural land, forest, lake, etc.). The loss of biodiversity contributes to food and energy insecurity; increases vulnerability to natural disasters, such as floods or tropical storms; and decreases the level of health worldwide. Plant biodiversity is at the basis of agriculture, as it allows for the production of plants, and consequently a broad diversity in terms of foods and feeds, contributing to the health and nutrition of the entire global population.

Italy is one of the European Countries with a broad biodiversity, and holds about the 37% of the total Euro–Mediterranean flora and fauna. Unfortunately, both are increasingly endangered and declining [11].

In this context, wild and managed bees play a fundamental role for two reasons: (i) they are themselves useful bioindicators for monitoring biodiversity levels, and (ii) due to their pollination activity, they play a key role in the biodiversity conservation, recovery, and restoration of degraded habitats and in the ecosystem services supply.

### 2.2. Bees and Ecosystem Services

Wild bees and reared honeybees are the main pollinators in most ecosystems [12,13]. Although not representing the most diverse or numerous pollinators [14], bees are the most effective biotic pollinating organisms. They are reliable and active because they search for flowers and identify the species that they prefer to visit in several different environments [15,16].

Bees comprise about 20,000 described species across seven recognized families [17]. In Europe, about 37–52% of bee species are included on the World Conservation Union Red List [18]. The importance of the role of bees at an ecosystem level cannot ignore the deep knowledge of ecosystem relations and services.

Many services essential to the survival, health, livelihoods, and human well-being are supplied by ecosystems [19]. According to Burkhard and Maes [20], ecosystem services (ES) are important contributors towards ecosystem structure and function towards human well-being.

**Figure 1:**
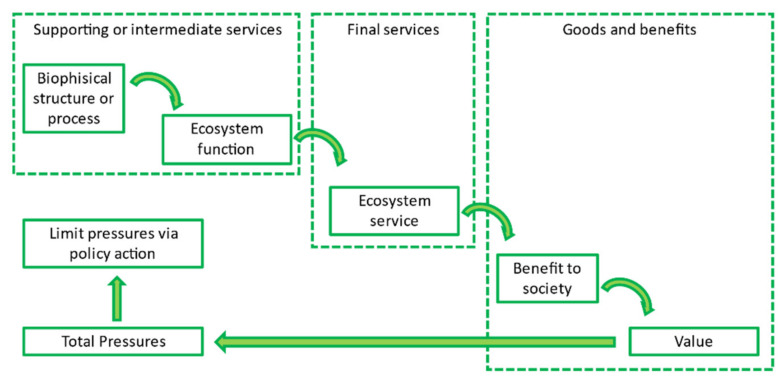
The cascade model (adapted from [24], modified).

Ecosystem services are well illustrated by the so-called “cascade model” [21,22,23], which describes the connections between biophysical structures (as a habitat type), processes (as primary production) of the ecosystem, and human well-being, as shown in Figure 1.

Ecosystem functions are the features or actions of the ecosystem that provide the capacity to deliver an ecosystem service (e.g., the forest or pasture can stock biomass). These characteristics are defined as supporting or intermediate services. The final ecosystem service is the product that can be harvested, like timber for the forest, or earned by the ecosystem, such as pollination. The final services directly contribute to human well-being through the benefits they support, such as health and safety.

Biodiversity has an essential role in the supply of ecosystem services, because it is associated with supporting and intermediate services [24].

In ecosystem management, it is necessary to recognize several ecosystem services provided by wild bees to humans, because every ecosystem is a set of interactions between different components [25]. In fact, sharp declines may occur in the provision of other ecosystem services when only one ecosystem service is considered [26]. Wild bees provide a large variety of ecosystem services to humans, e.g., pollination. Indeed, the pollination of flowering plants can be viewed as an essential ecosystem service of pivotal socio-economic importance. As remarked by Porto et al. [27], pollination systems are under increasing threat from anthropogenic sources/human activities, such as fragmentation of habitats, climate change, changes in land use, modem agricultural practices, the use of herbicides and pesticides, and the invasion of non-native plants and animals [27]. 

Close connections characterize wild bees and human systems. Humans depend on bees for ecosystem services, and bees depend on humans for their survival [28,29]. In this context, apiculture represents the close connection between these systems.

### 2.3. Beekiping, Bee Products, Floral Resources and Territory

Apiculture, also known as beekeeping, is defined as rearing and managing honeybees for commercial production, e.g., extraction, bottling, and sales of products of the beehive, such as honey, bee bread, bee venom, bee pollen, propolis, and royal jelly. 

Three products essential to the life of the bees originate from plants: honey, pollen, and propolis [30,31]. 

Honey, a supersaturated solution or semi-solid natural product, is produced from carbohydrate-containing exudates produced by plants, mainly from nectar sources containing sucrose, glucose, and fructose.

Pollen is the most important source of protein for bees, while propolis (bee glue) is a resinous material used by bees for both building and defensive purposes [32].

Other products are synthesized by the bees themselves, like beeswax, royal jelly, and venom. Royal jelly is produced directly by the bees from the hypopharyngeal and mandibular salivary glands of young worker bees and is used to feed the queen and larvae, the latter of which is only for a few days. Volatile and non-volatile compounds of royal jelly are influenced by plants that create the nectar and pollen collected by bees [33]. Royal jelly is considered a significant factor affecting the differentiation of larvae into the sub-populations of workers and queens as a consequence of the bioactive components there are, although how this development is achieved remains unclear [34]. 

Plant nectar contains water, sugar, and amino acids to attract pollinators, which find nectar sources in floral nectaries located at different places, for example, at the base of the ovary, stamen filaments, and petals, or on extra-floral nectaries on sepals or shoots [35]. As carbohydrates, free amino acids, and volatile compounds of nectar are important for the function of attraction, secondary metabolites are considered as a plant defence and have a less obvious benefit for pollinators [36].

In addition to nectar, bees obtain sugar substances from secretions produced by some tree species or by plant-sucking insects, mainly from the family *Aphididae*. Both the honey derived from nectar (blossom or floral honey) and that from secretions (honeydew honey) differ in their composition depending on the botanical and geographical origin [37]. Foraging bees fly over several flowering plants in the proximity of the bee colony. They only visit flowers of the same plant species; if the foraging material is sufficient, bees will remain faithful to the same species during further flights, and the target will be shared with other bees of the hive [38]. 

Unifloral honey originates from a single main source of nectar. Even if the apiary is placed in an area of a single main species nectar source, the unifloral honey from this species is not guaranteed, as bees might collect the nectar from other plants [39]. The botanical origin determines the physicochemical characteristics of unifloral honey [40] and influences consumer preferences. According to the main season of flowering of the nectar plant source, a unexhaustive list with examples of the botanical source for commercialized unifloral honey in Europe is shown in Table 2, while more than 100 botanical species are known to produce unifloral honey [41].

Honeydew honey originates from the collection of secretions from the living parts of the plant (e.g., the leaves) by bees or from sap-sucking insects. Honeydew is a component of some commercially appreciated unifloral honey of tree species, as in the case of chestnut (*Castanea sativa*), lime (*Tilia* sp.), and eucalyptus (*Eucalyptus* sp.) honey. In general, forest honey is a honeydew-predominant honey that comes entirely from forest plants. Depending on the botanical and geographical origin, forest honey can be dark coloured, stays liquid or viscous for a long time, and has a soft consistency [37]. Typical microscopic features of forest honey include honeydew elements such as fungi and algae, and a crystalline mass [42].

In Central Europe, honeydew honey originates mainly from fir species (*Abies alba*, *A. borisii-regis*) and spruce (*Picea abies*) trees [37], as single species honey, or even in a combination of both, as well as from *Salix* species [43]. In Mediterranean Countries, honeydew honey is originated from pines (among others, *Pinus halepensis* and *P. brutia*) or oak species, deciduous (e.g., *Quercus robur*, *Q. petraea*, *Q. frainetto*, *Q. hartwissiana*, *and Q. pyrenaica*), as well as evergreen ones (e.g., *Q. ilex* and *Q. suber*) [44,45]. Mediterranean pine forests of the southeast countries (e.g., Greece and Turkey) are locally infested by the scale insect *Marchalina hellenica*, which produces secretions from the absorption of pine phloem. Dark honey with high phenolic substances and antioxidant properties originate from these forests [46], affected by growth loss due to pest infestation [47]. Among other species, honeydew honey is also obtained from the native trees of *Mimosa scabrella* (Brazil), *Nothofagus solandri* (New Zealand), and *Quercus virginiana* (USA) [42,48].

On the other hand, multifloral honey originates from the nectar of a variety of flowering plants. Colour, consistency, smell, and taste depend on the type of flower involved and vary considerably [49]. A spring blossom honey meets the requirements of blossom honey (i.e., to have more than 60% nectar content), as well as nectar-supplying flowering plants that bloom in the spring months. As a floral resource, phenology might be altered by changes in climate and land use [50,51], and the botanical origin of multifloral honey varies throughout the year and between years.

Pollen, a protein and amino acid source for bees, is collected during visits on flowers of plants under the effect of a weak electrostatic field generated between flower (negatively charged) and bee body (positively charged) [52,53]. Pollen grains stored as pellets inside the hive originate from unique botanical taxon or different plant species, depending on the hive’s flowering resources. Bee pollen is appreciated as a natural food due to its nutritional and medicinal properties, while the physico-chemical, functional, and sensory properties of the product vary greatly according to the botanical origin [54].

Bees produce propolis as a defensive material by using resinous plant materials mixed with wax and other organic substances. Despite the variety of plants that secrete antimicrobial resins to protect the buds, young leaves, and wounded tissues, the number of known plant taxa used as a raw source for propolis is limited worldwide to a few dozen [31]. The main sources for propolis production are tree species of the genera *Populus* (e.g., *Populus tremula*, and *P. nigra*) and Betula for Europe, while in tropical and subtropical areas, Dalbergia and Acacia (*Fabaceae*), Macaranga (*Euphorbiaceae*), Mangifera and Rhus (*Anacardiaceae*), and Baccharis (*Asteraceae*) are known as resin resources for bees. As the collection of antimicrobial plant resins and their use in the nest architecture represent an energy-demanding activity for bees with no direct reward for the individual foraging bee, the term “social immunity” has been used to describe the function of propolis on colony health and resiliency [55].

The great variety of floral species involved in bee foraging might be insufficient to invert the worldwide bee decline [56], while intensification has led to a reduction of plant taxa, which are the nutrient resources for bees, and farmland bee-pollinated crops may be available to a large extent. Still, they represent single sources of nectar or pollen that are not always optimal for pollinators [57,58]. Under some specific climate conditions, the crop bloom does not overlap with the foraging periods of bees, affecting the reproduction and development of their vital needs.

## 3. Nutritional Value, Bioactive Components, and Beneficial Properties of Bee Products

An overview of the main components of bee products, their biological potentials, and health is here highlighted and contextualized as follows: (i) nutritional value of bee products; (ii) bioactive profile of bee products and their related beneficial properties; (iii) focus on honey considering health, biodiversity, and sustainability through a literature quantitative analysis; and (iv) bee products considered through exploring databases.

### 3.1. Nutritional Value of Bee Products 

The physicochemical characteristics, nutritional components, and bioactive compounds vary significantly according to the type of products, such as honey, beeswax, pollen, propolis, and royal jelly. Such traits can be related to various factors, including the bee type, floral source and geographical location of production, climatic conditions, seasonal factors, soil composition, and production process.

Honey is considered one of the most complex natural foodstuffs, and is defined as a natural sugar-saturated material. It is used as a food sweetener, complete food, nutraceutical agent, and medicinal supplement—it is composed of carbohydrates, proteins, lipids, amino acids, minerals, enzymes, and vitamins, as well as other constituents, such as enzymes, organic acids, carotenoids, vitamins, and aromatic substances. It is rich in flavonoids and phenolic acids, exhibiting various biological effects such as antioxidant properties.

Several studies have reported the proximate composition (*w*/*w*) of natural honey as follows: 80–85% carbohydrates (mainly glucose and fructose); 15–17% water; 0.3–0.4% proteins; 0.2% ashes; and minor quantities of organic acids, minerals, amino acids, vitamins, phenols, and pigments [59,60,61].

The carbohydrate content of honey consists of mono-, di-, and tri-saccharides, where floral type is a key factor in modulating this ratio. For instance, Tedesco et al. [62], by describing the composition of sugars in unifloral, multifloral, and some honeydew honey, identified more than 20 oligosaccharides in different varieties of honey, usually at relatively low concentrations. The protein contents consist of free amino acids and enzymes. The main amino acid observed in honey is proline [63,64].

Several studies have been carried out on the mineral content of honey [65,66]. Ribeiro et al. [67] reported how potassium and sodium constitute mostly 80% of the total minerals, while iron, copper, and manganese are rare in quantity. Moreover, trace elements have been used recently to identify different unifloral honeys [67].

Bee pollen contains bioactive compounds, including proteins, amino acids, lipids, carbohydrates, minerals, vitamins, and polyphenols [54,68]. Considering pollen, it has been reported that the bee pollen’s protein concentration varies between 10% and 40% (*w*/*w*), depending on the botanical origin. The recent work of Al-Kahtani et al. [69], studying the effect of the harvest season on the nutritional value of the bee pollen protein, reported that the highest contents of crude protein, and total and essential amino acids, were observed in bee pollen collected in the spring and winter seasons.

Propolis consists of resin (50%, *w*/*w*); wax (30%, *w*/*w*); essential oils (10%, *w*/*w*); pollen (5%, *w*/*w*); and other substances such as phenolic compounds, esters, flavonoids, terpenes, beta-steroids, aromatic aldehydes, and alcohols [70,71].

Royal jelly is mainly constituted by water, proteins, carbohydrates, and lipids, as well as trace amounts of minerals, vitamins, and phenols, which are present in small quantities [72]. Proteins represent the most abundant fraction when considering only the dry matter of royal jelly [34], and the majority of them (82–90%, *w*/*w*), the so-called Major Royal Jelly Proteins (MRJPs), are well studied [34]. As for the other bee products, it is well documented how royal jelly is naturally non-homogeneous, and its composition seems to be influenced by several factors such as seasonality, geographical conditions, floral differences, and species of honeybees [73]. For instance, it has been recently shown that climatic conditions influence the bioactive profile of Iranian honey [74]. This evidence demonstrated that royal jelly obtained from dry and arid regions has a higher nutritional value (i.e., higher 10-HDA and amino acids content), due to less rainfall and more aromatic and medicinal vegetation, in comparison with wet and moderate areas [74].

### 3.2. Bioactive Profile of Bee Products and Related Beneficial Properties

Bee products are natural sources of bioactive compounds with potential beneficial properties. Investigating the phytochemical differences is very important since the bioactive profile of bee products is responsible for their biological and therapeutic properties [75]. Several works have investigated the beneficial properties of bee products [76].

In the following, the bioactive contents of the main bee products of interest to the food industry and their related potential beneficial effects on human health are summarized.

#### 3.2.1. Honey

Honey is a food matrix with a complex composition that strongly depends on the bee species, environmental conditions, and botanical origin. As described above, it is composed mainly of carbohydrates (e.g., fructose and glucose, and di- and oligo- saccharides), but many minor components are also present, including bioactive compounds such as phenolic compounds, vitamins (e.g., vitamin B complex and ascorbic acid), enzymes (e.g., glucose oxidase, diastase, α-glucosidase, catalase, and acid phosphatase), organic acids (e.g., gluconic and citric acid), amino acids, peptides (e.g., defensin-1 and royal jelly protein isoforms), and minerals (e.g., potassium) [75,77,78]. Bioactive compounds are of great interest for their potential beneficial effects on human health. For this reason, honey has been used in traditional medicine as a natural remedy since ancient times, both for internal and external uses [79,80,81,82,83]. In particular, the beneficial properties of honey have been mainly associated with phenolic compounds, primarily flavonoids, and phenolic acids, which are the most abundant phytochemicals in honey [77]. Among the flavonoids, several of them (e.g., myricetin, kaempferol, quercetin, isorhamnetin, pinobanksin, rutin, and galangin), as well as flavones (e.g., luteolin, apigenin, tricetin, and chrysin) and flavanones (e.g., naringenin, pinocembrin, and pinostrobin), have been identified. At the same time, the phenolic acids in honey are represented by hydroxybenzoic acids (e.g., syringic acid, gallic acid, ellagic acid, protocatechuic acid, syringic acid, and p-hydroxybenzoic acid), hydroxycinnamic acids (e.g., chlorogenic, cinnamic, sinapic vanillic, caffeic, p-coumaric acid, and ferulic acids) and hydroxyphenylacetic acids (e.g., p-hydroxyphenylacetic acid) [78,84,85]. 

Although flavonoids and phenolic acids always remain the most abundant classes in honey, the phenolic profiles vary qualitatively and quantitatively according to the honey floral sources and geographical origin. In more detail, the multifloral honey composition is even more variable than for monofloral honey, as it presents several floral origins. For instance, an analysis of 16 honey samples showed that phenolic acids prevailed in some samples, while flavonoids were dominant in other honey. Moreover, in some kinds of honey, phenolic acids and flavonoids were present in the same percentages [84]. In another study, the total phenolic content ranged between 763 and 3508 mg GAE/ Kg among 21 different honey samples, thus pointing out the variability of honey due to its origin [85]. 

In different types of honey, the identification and quantification of phenolic compounds and other bioactive molecules has been of great interest in recent years for two main purposes: (i) honey traceability to identify specific marker compounds and to discriminate the floral origin of honey [85,86], and (ii) to select the honey with the best beneficial properties, as differences in the bioactive profile necessarily reflect the therapeutic properties [84]. 

Polyphenols are well known for their antioxidant and anti-inflammatory properties, and consequently play a key role in preventing noncommunicable diseases such as cardiovascular diseases, neurodegenerative disorders, cancer, diabetes, and obesity, which are included in the cluster of health conditions named “metabolic syndrome” [87,88]. A positive correlation between honey’s total phenolic content and its antioxidant activity has been observed in several studies, thus highlighting the role of phenols in determining the beneficial properties of honey [78,84,85,86,89]. For instance, it has been demonstrated that dark-colored honey (i.e., honeydew honey) has a higher antioxidant activity due to the higher content of flavonoids and phenolic acids [85,86]. A high positive correlation between the concentration of chlorogenic acid and the antioxidant activity in a monofloral Chilean honey has been observed [89]. However, in some cases, honey with the same total phenolic content did not exhibit the same antioxidant capacity, thus suggesting the presence of other non-phenolic origin antioxidants such as vitamin C [84]. 

The *in vitro* anti-inflammatory activity of phenolic acids and flavonoids from honey has been demonstrated in Malaysian honey extracts by evaluating their effect on TNF (tumor necrosis factor)–α activity and NO (nitric oxide) inhibition [90]. Moreover, recent studies have pointed out that phenolic compounds from stingless bee honey (e.g., the meliponina) also exhibit anti-inflammatory effects [91]. Due to its anti-inflammatory activity, a honey flavonoid extract has been even suggested as a potential preventive and therapeutic agent for the treatment of neurodegenerative diseases involving neuroinflammation, namely Alzheimer’s and Parkinson’s disease [92]. Notwithstanding this, other honey bioactive molecules have also shown immunomodulatory activities, for example, major royal jelly proteins [93], glycopeptides, and glycoproteins [94].

The anti-diabetic activity of honey has been previously reported and is associated with the presence of phenolic compounds and oligosaccharides with prebiotic effects [95,96]. 

Furthermore, the antimetastatic, antiproliferative, and anticancer effects of honey flavonoids and phenolic acids have been widely investigated in certain cancer cell lines such as breast, liver, and colorectal, and the potential mechanisms have been unraveled and extensively reviewed [97,98]. In addition, Malaysian jungle Tualang honey has been suggested as a natural cancer-alleviating agent and as a supplement to chemotherapeutic agents due to its ability to alleviate breast carcinogenesis in a rat model through modulation of the hematologic, estrogenic, and apoptotic activities [99].

Polyphenols are also known for their antimicrobial properties against pathogenic and spoilage microorganisms [100]. In this regard, phenolic extracts from monofloral Ulmo honey samples inhibited *Staphylococcus aureus*, *Streptococcus pyogenes*, *Pseudomonas aeruginosa,* and *Escherichia coli* [89]. Interestingly, honey samples have been effective even against drug-resistant bacteria, other than against reference and clinical strains, as previously reported by [84]. Furthermore, flavonoids also seem to be responsible for honey’s inhibitory activity against *C. Albicans* [101]. Also, it has been demonstrated that defensin-1 and H_2_O_2_ were accountable for the bactericidal activity of Revamil source honey, while methylglyoxal was the major bactericidal factor in manuka honey [102].Other characteristics of honey that also contribute to its antimicrobial activity are its high osmolarity, low water activity, low pH, and the presence of glycoproteins with high-mannose N-glycans and glucose oxidase, which convert glucose into H_2_O_2_ and gluconic acid [75]. Moreover, honey glycoproteins and glycopeptides have also been proposed as antiprotozoal agents against *Giardia lamblia* [103]. At the same time, an unidentified glyconjugate molecule has been identified as being responsible for the nematicidal activity of natural honey using *C. elegans* in a model system [104]. 

Finally, other therapeutic activities related to the biological activities described above (i.e., the antioxidant, anti-inflammatory, and antimicrobial activities) have been reported for honey, such as wound healing and its involvement in preventing liver, cardiovascular, and digestive disorders [75,77,105].

#### 3.2.2. Royal Jelly

Royal jelly has long been used in traditional medicine, and nowadays its interest as a functional food is growing due to its beneficial properties. Royal jelly’s health benefits have been attributed to its bioactive compounds, mainly proteins, peptides, lipids, polyphenols, and other minor active molecules [106].

Most of these compounds (82–90%, *w*/*w*), the so-called Major Royal Jelly Proteins (MRJPs), are considered among the principal bioactive components in royal jelly. Nine MRJPs have been identified, with MRJP1 accounting for more than 45% [34]. It has been demonstrated that MRJPs are involved in promoting lifespan, feeding, and fecundity in *Drosophila melanogaster*, mainly acting as antioxidants [107]. A similar anti-aging activity was previously reported for MRJP1 on *Drosophila* [108] and the nematode *Caenorhabditis elegans* [109] through the epidermal growth factor receptor signaling-mediated pathway. Moreover, hypocholesterolemic [110] and antihypertensive [111] activities have also been attributed to MRJP1. As for the other MRJPs, MRJPs 2 and 4 exhibited antimicrobial activities against bacteria, fungi, and yeast, being bound to their surface and thus damaging the microbial cell wall [112,113]. In addition, MRJP2 has also been reported as an antioxidant agent capable of increasing mammalian cell viability [112]. Interestingly, MRJP2 and MRJP2 isoform X1 have recently been suggested as a promising therapy for SARS-CoV-2 (severe acute respiratory syndrome coronavirus 2) infection and as anti-hepatic damage and anti-cancer agents [114,115]. MRJP3 exhibited potent immunoregulatory effects *in vitro* and *in vivo*, thus suggesting its involvement in royal jelly’s antiallergic and anti-inflammatory activity [93,116]. The immunomodulatory activity has also been attributed to MRJP1 and 2 [117,118], while MRJPs 2, 3, and 7 have been reported to possess a potential wound-healing bioactivity [119]. Finally, MRJPs increased the reproductive performance in female mice, thus promoting fertility [120].

Among the protein fraction of royal jelly, peptides have also shown biological activities. Notably, jelleines (I–III) and royalisin have been reported to possess an antimicrobial activity against yeast, fungi, and Gram+ and Gram− bacteria [121,122]. In addition, defensin-1 is another antibacterial peptide with a wound healing capacity, as reported by [123]. Furthermore, the authors of [124] demonstrated the neuroprotective effect of royal jelly peptides, thus underlying their role in relieving neurodegenerative diseases such as Alzheimer’s disease in the elderly. Lastly, small peptides with two to four amino acid residues derived from royal jelly protein have shown an antioxidant activity. Remarkably, those dipeptides that contained Tyr residues at the C-terminal (e.g., Lys-Tyr, Arg-Tyr, and Tyr-Tyr) exhibited the strongest hydroxyl-radical and hydrogen-peroxide scavenging activity [125].

Several biological properties of royal jelly have been associated with its lipidic fraction, mainly composed of C8-C10 hydroxy and dicarboxylic fatty acids. Among this fraction, trans-10-hydroxy-2-decenoic acid (10-HDA) has received great attention as it is the most characteristic compound of royal jelly, generally used as a quality and authenticity marker, and it exerts several beneficial effects [126]. Like for MRJPs, 10-HDA was reported to extend the lifespan of *C. elegans*, despite the mechanisms related to its longevity-promoting activity having been unraveled [127]. The involvement of 10-HDA in skin protection against ultraviolet radiation A and B induced photoaging has been elucidated [128,129]. 10-HDA has been even suggested as a potential medicine for rheumatoid arthritis and, along with 4-hydroperoxy-2-decenoic acid ethyl ester, as a therapeutic agent against atherosclerosis [130,131]. In addition, 10-HDA displayed an antimicrobial activity against Gram+ and Gram− bacteria and fungi [132,133,134]. Moreover, the neuroprotective, neurotrophic, and anticancer effects of 10-HDA have been also demonstrated [135,136]. 10-HDA, along with 3,10-dihydroxy-decanoic acid (3,10-DDA), possess a strong immunomodulatory capacity, while 10-HDA, 10-hydroxydecanoic acid (10-HDAA), and sebacic acid exhibit an anti-inflammatory activity [137,138,139,140,141,142]. Furthermore, estrogenic activity has been attributed to some fatty acids and sterols such as 10-HDA, 10-HDAA, trans-2-decenoic acid, 3,10-DDA, sebacic acid, and 24-methylenecholesterol, suggesting royal jelly as an anti-menopause agent [143,144]. 

Among the minor bioactive molecules, several flavonoids and phenolic acids have been previously detected in royal jelly, including chrysin, naringenin, and ferulic acid, and they have been partly related to its antioxidant activity [145,146]. Finally, adenosine monophosphate (AMP), AMP N1-oxide, and adenosine N1-oxide have been shown to be other minor active compounds of royal jelly with neurotrophic and anti-inflammatory properties [147,148]. 

#### 3.2.3. Propolis

Propolis is another bee product well known for its reported health benefits, and has been used as a natural remedy in traditional holistic medicine approaches for a long time. It is composed of resin, wax, essential oils, and pollen. It contains several bioactive compounds, including phenolic acids, flavonoids, esters, diterpenes, sesquiterpenes, lignans, aromatic aldehydes, alcohols, amino acids, fatty acids, vitamins, and minerals [76,149]. Nonetheless, the chemical composition of propolis is substantially variable, depending on the geographic origins, and climatic conditions, but mainly on the vegetation growing around hives (e.g., the floral source). Investigating the composition of different propolis is important because these differences directly affect their biological activity [150,151]. 

Polyphenols are perhaps the main constituents of propolis that exert beneficial effects on health, and propolis has been reported as the bee product containing the highest amount of phenolics [75]. Several flavonoids and phenolic acids have been detected in propolis, including naringenin, apigenin, quercetin, isorhamnetin, artepillin C, kaempferide, kaempferol, pinobanksin, pinocembrim, chrysin, galangin, and genistein, as well as p-coumaric, chlorogenic, and caffeic acids [151,152,153]. In particular, caffeic acid phenethyl ester (CAPE) and artepillin C, two significant constituents of propolis, have been previously ascribed among the valuable biologically active compounds in propolis for their several biological and pharmacological properties [154]. Even though different propolis samples have similar phenolic profiles, significant quantitative differences have been observed among propolis originating from different regions or bee species, and consequently, dissimilarities have been observed in the biological activities exerted. The available studies have shown a high positive correlation between the total phenolic content and the antioxidant activity of propolis, thus confirming that its antioxidant activity is mainly due to the polyphenols content [151,152,153]. For instance, pinobanksin-3-acetate has been reported as the major antioxidant component in French propolis extracts [155]. In addition, other reducing compounds that can contribute to the antioxidant activity of propolis are some carbohydrates, organic acids, nitrogen compounds, and vitamins [152]. Propolis has been reported to prevent oxidative stress in the brain, thus exerting neuroprotective effects due to phenolics such as the caffeic acid phenethyl ester (CAPE) [156,157]. For instance, it has been suggested that CAPE provides neuroprotection against cerebral ischemia injury through its antioxidant action [158]. In addition, pinocembrin, a flavonoid abundant in propolis, has shown a neuroprotective activity and thus has been suggested as a potential therapeutic agent for the prevention and/or treatment of Alzheimer’s disease and cerebral ischemia [159,160,161]. 

Propolis has also been reported to modulate immune responses [75]. For instance, Brazilian green propolis effectively improved immune function in aged mice due to phenolic compounds, and artepillin-C in particular [162]. CAPE also showed an immunomodulatory activity by inhibiting cytokine production and the proliferation of T cells, thus suggesting the use of propolis to treat allergic disorders such as asthma [163]. Moreover, CAPE was also reported as a potent anti-inflammatory compound, and therefore seems to be responsible for the anti-gingivitis and skin protective activity of propolis [164,165,166]. It has also been demonstrated that polyphenols from propolis, particularly from the red one, inhibited atherosclerosis progression in mice mainly through the modulation of inflammatory and angiogenic factors [167]. In particular, pinocembrin seems to be one of the bioactive compounds most involved in the cardioprotective effects of propolis [168]. Pinocembrin has been also reported to alleviate cognition deficits in diabetic mice by protecting neurons from inflammation injury [169]. It has been also suggested that polyphenols are not the sole substances involved in the anti-inflammatory activity. Vitamins, terpenoids, steroids, amino acids, and proteins could exert such an activity through different mechanisms that are still controversially cleared [149,152].

Several *in vitro*, pre-clinical and clinical studies have also investigated the anticancer activity of propolis [75]. The flavonoids and phenolic acids of propolis have been shown to possess dose-dependent antiproliferative and proapoptotic activities on several cancer cell lines such as gastrointestinal, lung, and breast cancer cell lines, as well as leukemia, lymphoma, and malignant melanoma cells. Among the phenolic compounds to which these activities have been attributed, there are caffeic acid and its esters (e.g., CAPE), chrysin, galangin, pinocembrin, pinobanksin, pinobanksin-3-*O*-acetate, pinobanksin-3-*O*-propanoate, pinobanksin-3-*O*-butyrate, and pinobanksin-3-*O*-pentanoate [170,171,172,173,174,175,176]. Furthermore, other bioactive compounds with a reported anticancer activity are cardanol and cardol [177]; two phenolic lipids isolated from Thai propolis, nemorosone and plukenetione A; two polyisoprenylated benzophenones isolated from Cuban propolis [178,179], and propolins (A, B, C, G, and H) and prenylated flavanones isolated from Taiwanese propolis [154].

It has also been shown that propolis has strong antimicrobial activities against Gram+ (e.g., *Streptococcus mutans* and *Lactobacillus plantarum*) and Gram− bacteria (e.g., *Escherichia coli*), as well as against fungi (e.g., *Fusarium* spp., *Aspergillus flavus*, *A. niger*, *Penicillium nordicum*, *P. expansum,* and *P. commune*) and yeasts (e.g., *Candida* spp.) [180]. The antibacterial activity of propolis could be related to the presence of phenolic compounds such as CAPE, pinocembrin, galangin, quercetin, rutin, and naringenin [75]. For instance, a moderate correlation between the antibacterial activity and the polyphenolic content of Argentine propolis has been observed [153]. Interestingly, in this work, the antimicrobial activity was correlated better with the pinocembrin content than with the total polyphenol content, thus highlighting the effectiveness of this flavonoid against bacteria [153]. In addition, an antibacterial activity has also been ascribed to propolis volatile compounds such as β-eudesmol, dihydroeudesmol, δ-cadinene, α-pinene, trans-β-terpineol, benzyl benzoate, nerolidol, spatulenol, ledol, farnesol, and guaiol [75,181]. Furthermore, an antiviral activity has been reported for some propolis constituents, such as CAPE and 3,4-dicaffeoylquinic acid against HIV-1 [182] and influenza A virus, respectively [183]. Propolis has traditionally also been used to treat gastrointestinal, gynecological, dermatological, and oral diseases thanks to its ability to inhibit pathogenic organisms (i.e., parasites, yeast-like fungi, and bacteria) [76,149]. Finally, the wound healing potential of propolis has also been reported and investigated, and has been attributed to its antioxidant, immunomodulatory, and antimicrobial activities described above [184]. 

#### 3.2.4. Bee Pollen and Bee Bread

Bee pollen and bee bread are perhaps the most underestimated among the bee products. Since ancient times, bee pollen has been used for its health-promoting properties, while bee bread remained unexplored for long time. Bee pollen is a mixture of carbohydrates, proteins, amino acids, lipids, fatty acids, carotenoids, phenolics, enzymes and coenzymes, vitamins, and minerals, many of which exert bioactive properties [75]. In particular, polyphenols, unsaturated fatty acids, phospholipids, and phytosterols in bee pollen have been studied for their biological activities [185]. Bee bread is a bee pollen-derived fermented product that presents a similar composition to bee pollen but with remarkable quantitative differences, mainly due to its fermentation process. For instance, bee bread has a higher amino acid, sugar, lactic acid, and vitamin content compared with bee pollen. Moreover, bee bread is easily digestible and more readily absorbed by humans as the pollen grain’s multi-layered wall is destroyed by the natural fermentation, making this product more biologically active [186,187]. However, as for the other bee products, the composition of bee pollen and bee bread is strictly influenced by the botanical origin and geographical and climatic conditions, which influence these products’ bioactive properties [187,188]. 

As these bee products are an excellent source of energy and nutrients, they have always been well known for their involvement in improving humans’ and animals’ physical and mental states [186]. Phenolic compounds are the most investigated bioactive molecules in these bee products. The phenolic profile of bee pollen and bread is mainly characterized by flavonoids (e.g., quercetin, kaempferol, isorhamnetin, myricetin, and rutin) and phenolic acids (e.g., cinnamic and coumaric acids), and it is strongly influenced by the botanical origin [189,190,191]. Several authors have demonstrated a good correlation between the phenolic content and the antioxidant potential of bee pollen and bread, suggesting the involvement of polyphenols in many of the biological properties of these products [189,192,193]. An *in vivo* study showed that feeding bee bread to mice resulted in a significant improvement in their antioxidant effect compared with bee pollen-fed mice, highlighting the greater bioactivity of the bee bread [194]. The antioxidant activity of bee pollen and bee bread plays a key role in preventing other diseases in which free radicals are implicated, such as inflammation and cancer. The bee pollen ethanol extracts’ analgesic and anti-inflammatory effects have been attributed mainly to the flavonoids content [195,196]. Flavonoids, mainly myricetin, have also been associated with bee pollen’s immunomodulatory and are related to its anti-allergic activity [197]. In another study, the anti-allergic activity of bee pollen was associated with polyphenolic compounds and unsaturated fatty acids (e.g., malonic acid and α-linolenic acid) [198]. Bee pollen and bee bread have also been tested for their anticarcinogenic activity on several cell lines [185]. In that study, the authors suggested that the anticarcinogenic potential could be attributed to unsaturated fatty acids (e.g., linoleic, linolenic, and arachidonic), phytosterols (e.g., β-sitosterol), triterpene compounds like oleanolic and ursolic acids, and polysaccharides, as well as polyphenols. In another study, it was shown that the steroid fraction of the chloroform extract from bee pollen of *Brassica campestris* L. induced cytotoxicity in prostate cancer PC-3 cells, probably triggering apoptosis, suggesting that this fraction could represent a promising candidate for the treatment of advanced prostate cancer [199]. In addition, it has been also observed that bee pollen and bee bread could be helpful dietary supplements for treating chemotherapy side effects [186]. Bee pollen and bread also possess a strong antimicrobial activity, possibly due to their phenolic content, but also due to the activity of the glucose oxidase secreted by honey bees [75]. However, the antimicrobial activity of bee bread could also be attributed to the reduction of pH following the anaerobic fermentation process carried out by lactic acid bacteria [186]. Several studies have indicated that bee pollen is more active against Gram+ (e.g., *S. aureus* and *S. pyogenes*) than against Gram− bacteria (e.g., *P. aeruginosa* and E. *coli*) [200,201,202]. This tendency was also reported for both bee pollen and bread [203]. Bee pollen and bee bread have also been reported to exhibit an antimicrobial efficiency against fungi and yeasts like *Aspergillus niger* and *Candida albicans* [192,204]. 

Due to all these biological activities, bee pollen and bee bread have also been related to preventing many health pathologic conditions. For instance, bee pollen and bee bread exhibit cardioprotective effects due to their anti-atherosclerotic and hypolipidemic activity, and this has been mainly ascribed to the presence of polyphenols, but also to unsaturated fatty acids like α-linolenic acid (ω-3), phospholipids, and phytosterols [75,205,206,207,208]. Notably, α-linolenic acid is a precursor of prostaglandin-3, a major platelet aggregation inhibitor [75]. Bee pollen and bread have also been reported as hypoglycemic agents with an antidiabetic potential due to the presence of antidiabetic compounds like flavonoids, alkaloids, saponins, sterols, steroids, tannins, and sugars, which are present in pollen [208,209,210,211]. Moreover, the hepatoprotective effects of bee pollen have been tested *in vivo* using animal models, thus suggesting the use of bee pollen in the treatment of hepatocellular pathologies [185,212]. Furthermore, the ability of bee pollen to protect the damage induced to some organs (e.g., liver and kidney) seems to be related to the presence of antioxidant molecules such as phenols [71]. Finally, the same antioxidant compounds have been also associated with the detoxifying activity of bee pollen in cases of poisoning by organic compounds or drugs [213,214].

### 3.3. Focus on Honey between Health, Biodiversity, and Sustainability: Literature Quantitative Analysis

An overall picture of the literature existing in the research field of honey and the relationships existing between this food matrix, health, biodiversity, and sustainability has been investigated throughout a bibliometric analysis. The aim was to verify the relevance and impact of the research on this area, as a comprehensive bibliometric analysis might reveal information regarding the main research topics, trends, and research impacts. In July 2021, a search was conducted in the Scopus database for honey and health research. Bibliometric data were extracted from the Scopus online database (https://www.scopus.com/home.uri, accessed on 6 July 2021) using the search string TITLE-ABS-KEY (honey* AND health*). This search strategy identified publications that mentioned the relevant words or their derivatives in the title, abstract, or keywords. As a result, the following parameters were assessed: publication year, publication count, authorship, institution, country/region, and document type.

The “Analyze” and “Create Citation Report” functions of the Scopus web online platforms were used for the basic analyses. Bibliographic data were recorded, including the publication year, publication count, subject area, countries/regions, institutions, authorship, and document type. The search returned 5134 publications covering the period between 1915 and 2021. 

**Figure 2:**
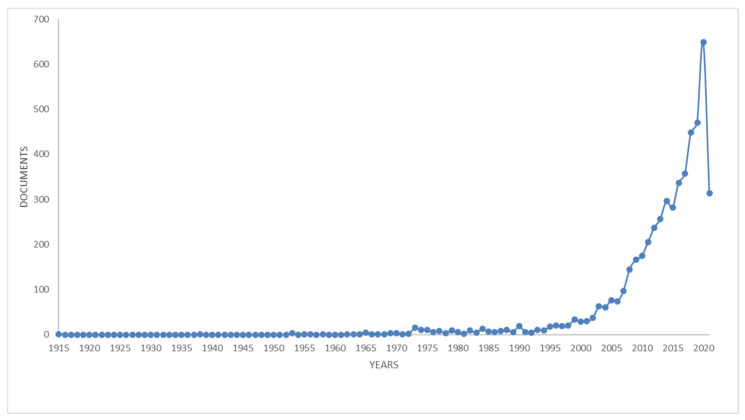
Publication trends of the relationships between honey and health research, displayed as a cumulative function (bibliometric data were extracted from the Scopus online database).

The publication trends of the relationships between honey and health research are reported in Figure 2. The first paper on this topic was published in 1915 by an anonymous author and concerns extra-floral nectaries [215].

The most recent work was published by Papa et al. [216] and studied the acute and chronic effects of titanium dioxide (TiO_2_) particulate matter on honeybee gut microbiota under laboratory conditions. Acute and chronic oral administration of ultrapure TiO_2_ particulate matter to adult bees altered the bee microbial community; therefore, airborne particulate matter may represent an additional risk factor for honeybee health, promoting sublethal effects against the gut microbiota [216]. The second most recent paper was a work by Sharif et al. [217] that, by looking for new, non-invasive methods to monitor the health status of the colony, introduced new features for classifying beehive audio samples using the soundscape indices [217]. In 2021, Nicewicz et al. [218] outlined the antioxidant capacity of honey from an urban apiary and honey from a rural apiary [218]. The most cited work was a paper published in the *Annual Review of Ecology and Systematics* in 1998 by Kearns et al., [219] focused on conserving plant−pollinator interactions.

**Figure 3:**
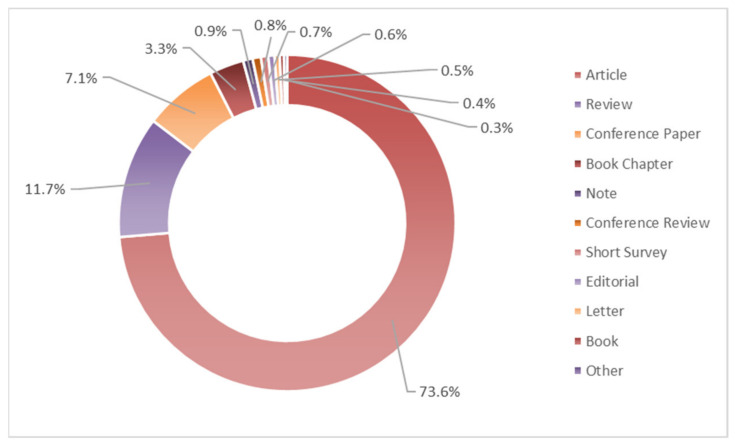
Distribution of documents by type (bibliometric data were extracted from the Scopus online database).

The types of documents related to the 5134 publications retrieved were distributed as reported in Figure 3. “Article” accounted for 73.6%, followed by “Review” (11.7%), “Conference paper” (7.1%), and “Book chapter” (3.3%).

**Figure 4:**
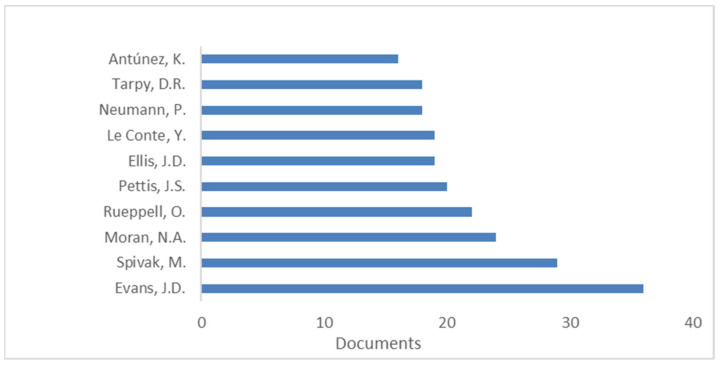
Most productive authors (bibliometric data were extracted from the Scopus online database).

Figure 4 reports the most productive authors. Evans J.D. (*n* = 36) was found to be the most productive author. His most highly-cited paper (cited 234 times) in the current dataset was a paper focused on individual and communal disease barriers in honey bees; the authors remarked how human efforts to maintain healthy colonies intersect with similar efforts by the bees, and how bee management and breeding protocols can affect disease traits in the short and long term [220].

**Figure 5:**
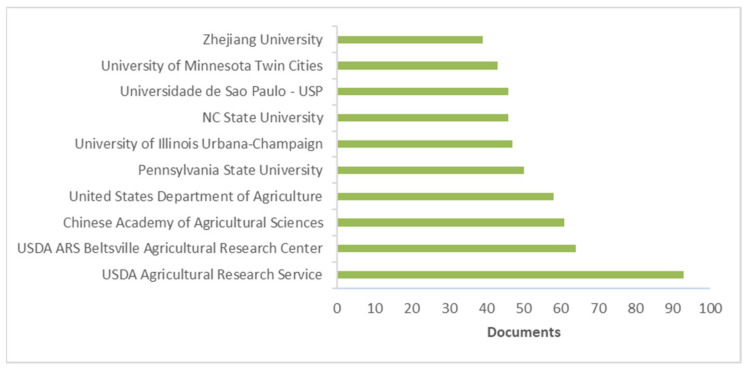
Most productive institutions (bibliometric data were extracted from the Scopus online database).

**Figure 6:**
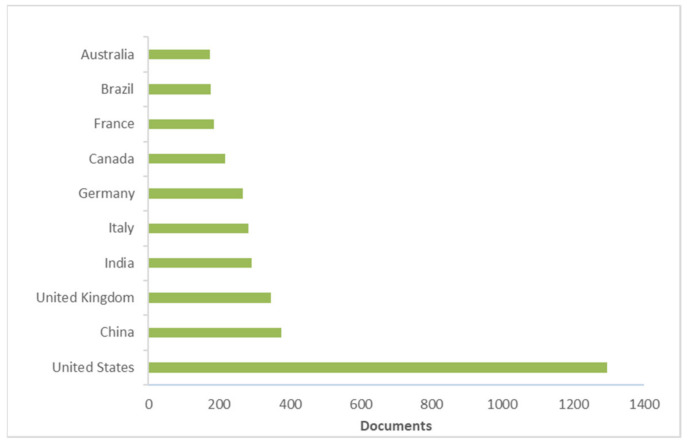
Most productive countries/territories (bibliometric data were extracted from the Scopus online database).

Figure 5 and Figure 6 show the most productive institutions and countries/territories, respectively.

The most productive institution was the USDA Agricultural Research Service of the United States (Figure 5). Its most highly cited paper was carried out by Chen and Siede [221] and considered the research of honeybee viruses. Regarding countries/regions (Figure 6), the most productive was the United States (*n* = 1296), followed by China (*n* = 376) and the United Kingdom (*n* = 346). For the United States, the most cited paper was the work of Kearns et al. [219], as previously mentioned. The most recent work was published by Milone and Tarpy, which evaluated the effects of developmental exposure to pesticides in wax and pollen on honeybee (*Apis mellifera*) queen reproductive phenotypes [222].

Addressing the search towards biodiversity, a search was carried out using the string: TITLE-ABS-KEY (honey* AND health* AND Biodiversity*). For further bibliometric analyses and additional processing, the “full records and cited references” were exported to VOSviewer software (version 1.6.16, www.vosviewer.com, accessed on 6 July 2021). The VOSviewer software (v.1.6.16, 2020) [223,224,225] analyzed the terms/words used in the titles and abstracts of publications by breaking down the paragraphs into words and phrases, linking them with the citation data of the publications, and visualizing the results in the form of a bubble map using a term map with the default settings. To simplify the bubble map, words/terms that appeared in at least five of the publications were analyzed and visualized. Of the 1578 keywords, 44 met the selected threshold, and 2 of them were manually excluded.

The search returned 91 publications covering the period from 1999 to 2021. The oldest publication was by Kevan [226], on pollinators as bioindicators of the state of the environment—their populations and diversity to monitor environmental stress brought about by introduced competitors, diseases, parasites, and predators through chemical and physical factors, particularly pesticides and habitat modification. On the other hand, the work of Popova [227], published on phytomedicine in 2021, comprehensively summarized and discussed the available data about the chemical composition of propolis from the stingless bee species (Meliponinae) of the Americas, Asia, and Australia, by giving a phytochemist’s guide through the jungle of tropical biodiversity. It is worth mentioning the most cited work: the research of Engel et al. [228], which, by investigating the role of honeybee’ gut symbionts for colony health and nutrition, opened the door for the importance of functional diversity within the simple gut microbiota of the honey bee [228]. These authors remarked how a honey bee can serve as a model for understanding more complex gut-associated microbial communities.

**Figure 7:**
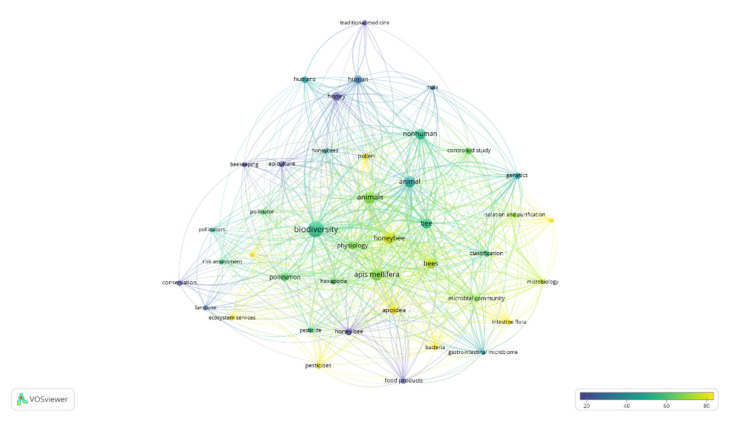
Term map of the relationships of honey, health, and biodiversity research. Bubble size represents the number of publications. Bubble color represents the citations per publication (CPP). Two bubbles are closer to each other if the terms co-appeared more frequently (bibliometric data were extracted from the Scopus online database and elaborated by VOSviewer software).

A total of 42 terms were derived from the quantitative literature research on publications, and they are visualized as a term map in Figure 7. The top recurring terms are shown in Table 3.

Narrowing the search, the search string TITLE-ABS-KEY (honey* AND health* AND biodiversity* AND sustainability*) returned seven publications.

Interestingly, the most recent work by Theodoridis et al. [229] presented and described the FoodOmicsGR_RI state-of-the-art facilities, using the unique, well-characterized sample sets obtained from precision/experimental farming/breeding including honey, along with more than 20 complementary scientific disciplines, moving towards the importance of sharing and data accessibility [229]. On the other hand, the work of Iatridou et al. [230] highlighted the importance of mapping the teaching of honeybee veterinary medicine in the European Union and European free trade area.

Among these results, the work of Arafah et al. [231] proposed and developed MALDI–MS profiling to address honeybee health status under bacterial challenge through computational modeling, while Edwards and Dixon [232], in 2016 published a paper regarding negotiating the conflict between humans and honeybees towards an ecological city in the journal of *Society and Animals*.

### 3.4. Bee Products: Exploring Databases

Nowadays, the overall goal is to link bioresources, platforms, and data repositories to food composition and dedicated databases, combining integrated and multidisciplinary datasets and developing research infrastructures while sharing data tools.

An example is given by the Bee Informed Partnership (BIP) platform [233], an open-access platform that represents an important service to beekeepers, researchers, and the general public in the United States. This platform provides a BIP database overview, a colony loss interactive map displaying nearly a decade of colony loss for each state, and management survey metrics. Moreover, it includes sentinel apiaries collecting data on hive health, the USDA Animal Plant Health Inspection Service, and a virus map. The Animal and Plant Health Inspection Service (APHIS) State reports review results from random samples of honeybee colonies. More information about this is available on the National Survey of Honeybee Pests and Diseases, available on the APHIS website [234]. The HoloBee Database v2016.1 [235,236], by USDA, it is also worth mentioning. It includes Hols Bee-Barcode, a non-redundant database of taxonomically informative barcoding loci for all viruses, bacteria, fungi, protozoan, and metazoans associated with honeybees (*Apis* spp.). Furthermore, HoloBee-Mop, a database comprised mostly of chromosomal, mitochondrial, and plasmid genome assemblies to aggregate as much honey bee holobiont genomic sequence information is an important resource for research in the area of interest.

Another example of an important platform is the Apiservices platform [237], which involves 131 countries and a 30-year long history, with the aim to offer beekeeping development services, such as supporting institutions through the integration of beekeeping in a multidisciplinary project; to inform and disseminate information throughout conferences and material such as photo, articles, reports, and educational packages for specific training purposes and conferences; and to innovate throughout market studies, website creation, and bench tests. 

In the perspective of the interoperability and sharing of data, a technical round table report on honey authentication published in 2018 by the European Commission [238] outlined how there is the need for infrastructures to tackle honey adulteration, covering the following four main areas: regulation, databases, quality assurance tools, and networking. In particular, databases populated by authentic samples represent important tools for honey authentication; it was remarked how a clear definition is required on what exactly is defined as “authentic honey”, and how databases storing information of compositional characteristics on honey should be representative, trustworthy, and accessible. Moreover, it is of utmost importance to share metadata, including information regarding botanical/geographical origin, bee species, season/year of production, storage practices, bee feeding practices, processing characteristics, blending practices, composition, and filtering manufacturing. 

The development of specialized databases of components with nutritional and nutraceutical properties at a national and European level represents a current challenge for better exploring the relationship between food, nutrition, health, and the environment [239]

Food Explorer, an innovative interface for finding food composition data, allows for simultaneously searching information from most of the available databases from the European Union (EU) Member States and from Canada, the United States, New Zealand, and Japan. Searching, for example, when searching “bee products” as “exact string”, and selecting all 39 databases, 27 records were retrieved, all of which referred to honey. In contrast, when searching as “all word”, more records, namely 218, were be retrieved, including all food products and recipes siting honey as an ingredient, such as breakfast cereals, cereal bars, cookies, nuts, and salad dressed [240,241]. 

Moreover, in the Italian Dietary Supplement Label Database [242], using a faceted search technique and faceted classification system that classifies each information element along multiple explicit dimensions, six dietary supplements belonging to the category of products such as “bee-produced formulations” [A03SQ] were present. Among them, four products contained “royal jelly” as the main ingredients, and two products contained “other edible apiculture products” as the main ingredients, such as extracts of propolis. The ingredient “royal jelly” was indicated using the facet [F04.A0CVG], whereas the ingredient “other edible apiculture products” was indicated using the facet [F04.A0CVF]. Moreover, the ingredient “royal jelly” was present in another category of dietary supplements, namely “mixed supplements/formulations” [A03TC], indicating any type of supplements combining different principles without a substantial prevalence of one. 

## 4. Case Study the “Cellulose Park” in Rome, Italy: Expression of the Interconnection between Health, Biodiversity, and Sustainability

The suburban area of Rome (Italy) is surrounded by forest plantations of Mediterranean tree species planted during the last century in the countryside, the so-called Agro Romano, characterized in the past by extensive pastures and few natural vegetation remnants. Being developed mainly in the south-western and litoral part of the area surrounding the city, this forest green belt is protected through natural reserves and parks for public enjoyment. It represents a buffer zone of increased biodiversity between the inner urban area of the about three-million inhabitants in the city and the intensive agricultural areas of the countryside.

**Figure 8:**
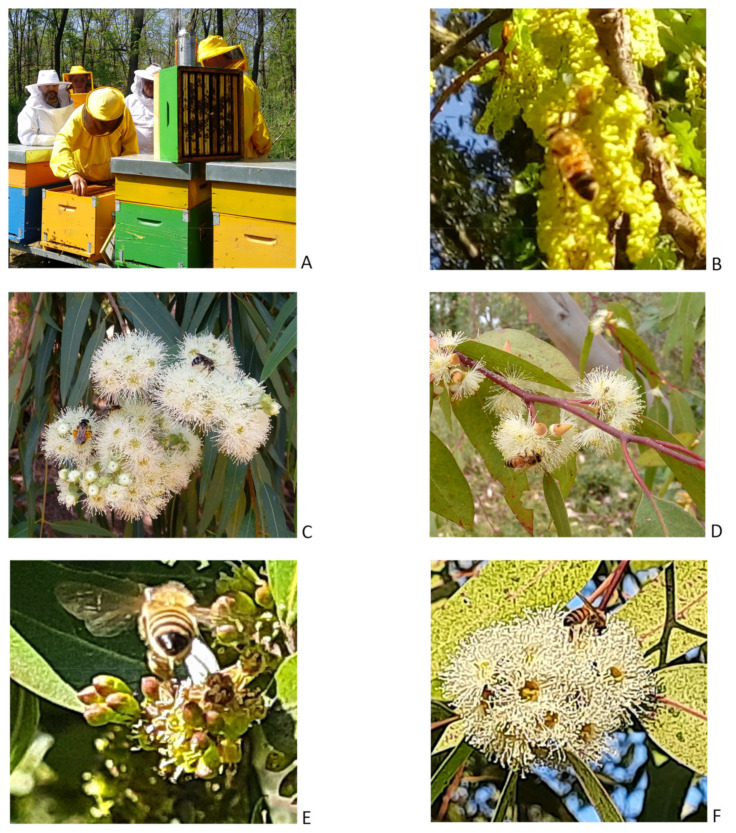
Beekeeper’s activity (**A**) and some plant nectar and pollen sources for bees and wild bees in the “Cellulose Park” of Rome (Italy): (**B**) *Quercus pubescens* (spring), (**C**) *Angophora floribunda* (summer), (**D**) *Eucalyptus* sp. (summer), (**E**) *Rhamnus alaternus* (spring), and (**F**) *Eucalyptus grandis* (winter).

“Cellulose Park” originated from research activities on several tree species (e.g., *Eucalyptus*, *Pinus*, *Cupressus*, *Prunus*, *Juglans*, and *Cedrus*), mainly as provenance trials for forest reproductive materials purposes, and is now protected under regional law. In 2018, beekeepers were allowed to establish their apiaries (see Figure 8), with a group of about 80 hives, on a forested area of about 50 hectares, surrounded by grassland areas, pastures and some limited extensions of intensive crops (maize). From 2019, the littoral area of Rome was infected by the pest *Toumeyella parvicornis* (tortoise scale of the family *Coccidae*), a phloem sap sucker mainly on *Pinus pinea* (Italian stone pine). 

Honey samples were collected and compared between the years and with samples of monofloral honeys of different botanical origins.

### 4.1. Sample Description

Five samples of multifloral honey were collected during the years 2019, 2020, and 2021 in “Cellulose Park” from hives located inside the forest plantations. Their main characteristics are reported in Table 4. Three additional samples of unifloral (*Castanea*, *Tilia*, and *Ailanthus*) honey, originating from the surrounding area outside of the park, were added to the analysis of the chemical elements for comparison. All samples were collected from beehives managed under the same conditions by beekeepers. The samples were stored at 20 °C in the dark until analysis for a maximum time of 36 months. Samples varied markedly for colour, with the *Ailanthus* and *Tilia* honey being the palest, and *Castanea* and multifloral honey generally being the darkest. Moreover, the description and classification system, FoodEx2 (Revision 2), developed by the European Food Safety Authority (EFSA), was applied for coding products in line with data harmonization and standardization procedures [243]. Because of the explorative character of the study, pollen analysis was not carried out for each of the above-mentioned honey samples, but was available for reference only on two samples of honey, collected in “Cellulose Park” in 2018 and 2020. 

### 4.2. Methodology

Pollen analysis was carried out using optical microscope for the determination of the botanical origin and number of pollen grains according to the national standards regulation (UNI 11299:2008). Macro- and micro-elements and heavy metals were determined in the filtered extracts, using inductively coupled plasma optical emission spectrometry (ICP-OES) equipped with an ICAP 6100 (Thermo Scientific, Waltham, MA, USA) spectrometer, following wet acid digestion method (5 g sample, 20 mL of 65% HNO_3_, 140 °C). The analytical parameters of ICP-OES were as follows: applied power of 1.3 kW, nebulizer flow rate of 0.8 L min^−1^, plasma gas flow of 15 L min^−1^, and an auxiliary gas flow of 2.0 L min^−1^. Before the analysis, the ICP-OES instrument was calibrated with a blank and four multi-element standard solutions. Elemental profiles in the samples were analysed with the principal component analysis (PCA) package using the base R function.

### 4.3. Results

The pollen analysis of the samples of honey evidenced a slight increase in plant diversity (2018 vs. 2020), as the total number of taxa shifted from 31 to 38, with an increasing number of *Fabaceae* taxa (e.g., *Trifolium*, *Genista*, *Hedysarium*, and *Lotus*), a decrease of *Rosaceae* (e.g., Crataegus and Prunus, Malus), and several new taxa (e.g., *Arecaceae*, *Betulaceae*, *Boraginaceae*, and *Cupressaceae*). The main change between the samples could be related to the presence of honeydew: from absent (2018) to a high content (2020). This could be related to the *Toumeyella* infestation of the *Pinus pinea* trees. The change in “Cellulose Park” honey was also evidenced by the colour difference of the samples, which turned from a pale colour in the spring sample of 2019 (BLO-19SP) to dark ones in the subsequent samples of honeydew honey, beginning from the 2019 summer sample (HDW-19SU) to the other samples collected in the years 2020 and 2021. 

Data on chemical elements of honey samples are shown in Table 5.

**Figure 9:**
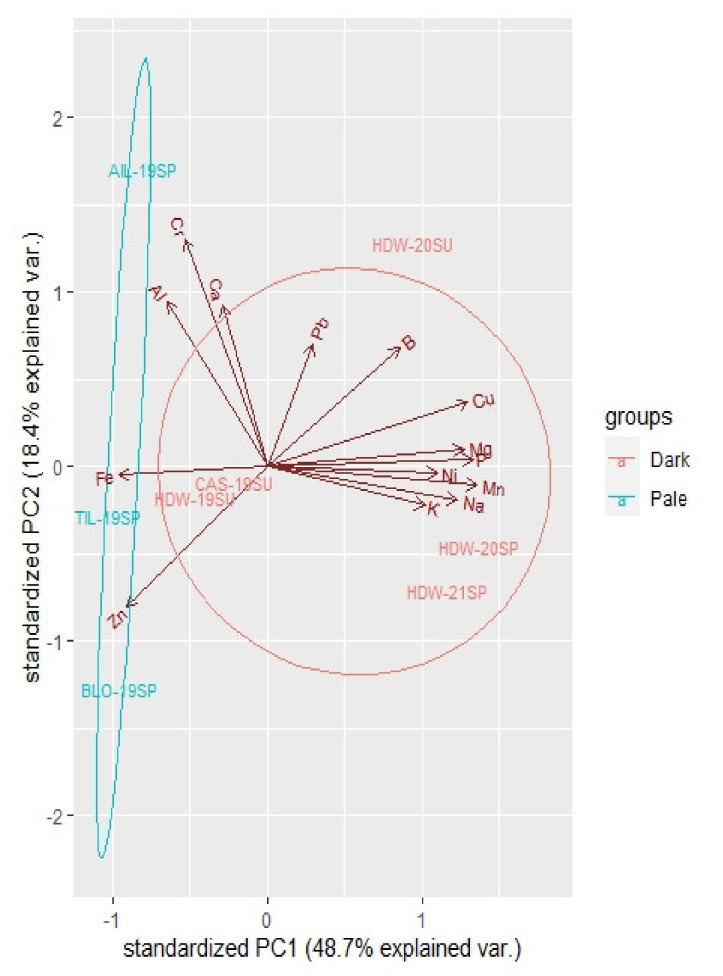
Principal component analysis (PCA) plot calculated from values for chemical elements in the honey samples.

Cadmium and arsenic were below the detection limit. The 2019 spring honey of the “Cellulose Park” sample (BLO-19SP) evidenced the highest zinc content and the lowest content of potassium and magnesium, which made it, to some extent, similar to the monofloral samples of *Ailanthus* and *Tilia* collected during 2019 (AIL-19SP and TIL-19SP samples). On the other hand, some honeydew honey samples (HDW-19SU, HDW-20SP, HDW-20SU, and HDW-21SP) showed a high potassium, magnesium, and manganese content, in a more similar content to the monofloral honey of *Castanea* (CAS-19SU). The PCA plot shown in Figure 9, which reflects the mentioned chemical samples characteristics, shows the differentiation between blossom and honeydew honey samples on the axis PC1, according to the year of honey extraction and honeydew in increasing importance for the “Cellulose Park” samples, with the 2019 spring honey (BLO-19SP) on the left side of the diagram and honeydew honey represented in the middle (2019 sample), and the right of the plot (2020 and 2021 samples) indicating the group of dark honey. Thus, honeydew samples evidenced higher values for phosphorus, potassium, nickel, and sodium, and, as mentioned before, magnesium and manganese.

The reported case study highlighted a possible profound change in the ecosystem due to the introduction of a new insect, which is directly reflected in the honey collection by bees. While further confirmation is needed on larger areas and on a larger number of samples, the chemical analysis of the honey samples seemed to reflect, to some extent, the change. Bees’ activity can be used in a similar way to assess and track the changes in the quality of agricultural ecosystems [244]. From the point of view of beekeeping, honeydew of forest plants represents a source of nourishment for bees in early spring, especially when adverse weather conditions may have compromised the first blooms, but also in summer and autumn, when, for example, the Mediterranean environment offers less nectar [45]. In the last years, the spread of *Toumeyella* from the coastal areas near to the investigated location to the inner parts of peri-urban area of the city of Rome increased the average honey production by 30% compared with previous years. However, the constant presence of honeydew compromises the production of monofloral honey (for the area where plants like Acacia, Lime, and *Ailanthus* are present), and little is known about nutritional requirements of bee species in order to develop diverse and nutritionally balanced plant communities [58]. In fact, the proliferation of the scale beyond a specific limit on pines located in the peri-urban area of the city of Rome, due to the development of several generations during the same year by mild winter conditions favoured by climate change, led to the death of the trees [245]. Honeydew collection activity by the bees under these conditions will therefore probably end soon. 

The analysis of the elemental composition of honey evidences its nature of a valid and sensitive matrix to detect the amounts of heavy metals present in the environment. The information obtained offers the possibility to acquire data on the contaminants present, even in trace amounts. Thus, hive products might be valid indicators for the quality and health of the surrounding environment and for changes induced by biotic and abiotic factors that impact the sustainability of agricultural production and biodiversity conservation in peri-urban areas.

## 5. Conclusions and Future Remarks

A multidisciplinary approach based on the interconnection of food, health, and biodiversity allowed for better defining and obtaining a product of a high quality that reflects the biodiversity of the territory. This may be the basis for further detailed research on the relationship between plant and animal biodiversity and human health. Protection of bees is a priority in the era of global climate change and loss of biodiversity. The literature analysis revealed that the nutrient and bioactive profiles of bee products are strictly dependent on the bee species, and on the geographical and botanical origins, thus underlying the vital link between the bioactive profile of bee products and the ecosystem. Therefore, a range of diverse floral resources is a key factor in sustaining healthy bee populations and biodiversity on a landscape scale.

As remarked in the case study presented, hive products can be viewed as new indicators of ecosystem and biodiversity. 

At the same time, recent studies are addressing the use of nanotechnologies [246,247] that could improve the functional properties of honey. Tang et al. [248] showed how honey-loaded alginate/PVA nanofibrous membrane could be used as a potential bioactive wound dressing. On the other hand, Hooven et al. [249], in a review published in 2019, discussed the properties, benefits, and types of nanotechnology-based pesticides; the propensity of bees to collect such particles; and potential impacts on bee pollinators. The authors remarked in their conclusion how agricultural and scientific communities should make every effort to investigate not only potential benefits, but also potential risks from new pesticide formulation technologies [249]. The use of pesticides and their impact on the environment could pose a serious threat to biodiversity, which depends also on the bees’ activity and wellbeing. 

## Figures and Tables

**Table 1:** Main bioindicator features (based on [5,6,7,8]).

Bioindicator Number	Bioindicator Features
1	Sufficiently sensitive to provide an early warning of change and relevant to ecologically significant events
2	Distribution over a broad geographical area
3	Applicable on a large scale
4	Attitude of providing a continuous assessment over a wide variety of stress
5	Relatively independent of sample size
6	Easy and cost-effective data collect and analysis
7	Able to differentiate between natural cycles and cycles induced by anthropogenic stress

**Table 2:** A not exhaustive list of the botanical origin (genus with the related species) for commercialized unifloral honey in Europe concerning the flowering season of the plant source.

Plant Habit	Genus	Spring	Summer	Autumn	Winter
**Trees**	*Eucalyptus*	*E. occidentalis*	*E. camaldulensis, E. viminalis*	*E. globulus, E. gomphocephala*	*E. globulus, E. grandis*
	*Citrus*	*C. limon, C. deliciosa, C. sinensis*	*C. limon, C. sinensis*	*C. limon*	*C. limon*
	*Prunus*	*P. domestica, P. spinosa, P. dulcis, P. armeniaca*			
	*Malus*	*M. sylvestris*			
	*Robinia*	*R. pseudoacacia*			
	*Ailanthus*		*A.altissima*		
	*Castanea*		*C. sativa*		
	*Tilia*		*T. platyphyllos, T. cordata*		
					
**Shrub/Climber**	*Hedera*			*H. helix*	
	*Rosmarinus*	*R. officinalis*	*R. officinalis*		
	*Thymus*		*Th. vulgaris, Th. capitatus*		
	*Arbutus*			*A. unedo*	
	*Erica*	*E. arborea*			
	*Rhododendron*		*Rh. hirsutum, Rh. Ferrugineum*		
	*Calluna*			*C. vulgaris*	
**Crops/Herbaceus Plants**	*Brassica*	*B. napus*	*B. napus*	*B. napus*	
	*Helianthus*		*H. annuus*		
	*Taraxacum*	*T. officinale*			
	*Lavandula*	*L. stoechas, L. angustifolia*	*L. angustifolia*		

**Table 3:** The top-recurring terms on the relationships between honey, health, and biodiversity research (bibliometric data were extracted from the Scopus online database and elaborated by VOSviewer software).

Terms	Occurrence	Total Link Strenght
Biodiversity	51	337
Animals	29	311
*Apis mellifera*	27	238
honeybee	25	225
Animal	25	276
Nonhuman	22	211
Bees	21	238
Bee	21	240
honey	15	87
*Apoidea*	14	118
Human	13	98
Pollination	13	116
Physiology	13	170
Honey bee	11	74
Controlled study	10	119

**Table 4:** Honey samples main characteristics: botanical origin, collecting season, and geographical origin (inside “Cellulose Park” or in the surrounding area).

Honey Sample	Botanical Origin	Honey Extraction Season	Geographical Origin	Colour	FoodEx2 (Revision 2) Code
AIL-19SP	*Ailanthus*	spring	surrounding area	pale	A033K#F10.A0F2Z Honey, monofloral, QUALITATIVE INFO = Pale/light colour
TIL-19SP	*Tilia*	spring	surrounding area	pale	A033K#F10.A0F2Z Honey, monofloral, QUALITATIVE INFO = Pale/light colour
BLO-19SP	Multifloral, blossom	spring	Cellulose Park	pale	A033L#F10.A0F2Z Honey, polyfloral, QUALITATIVE INFO = Pale/light colour
HDW-19SU	Multifloral, honeydew	summer	Cellulose Park	dark	A033L# F10.A0F2Y Honey, polyfloral, QUALITATIVE INFO = Dark
HDW-20SP	Multifloral, honeydew	spring	Cellulose Park	dark	A033L# F10.A0F2Y Honey, polyfloral, QUALITATIVE INFO = Dark
CAS-19SU	*Castanea*	summer	surrounding area	dark	A033K#F10.A0F2Y Honey, monofloral, QUALITATIVE INFO = Dark
HDW-21SP	Multifloral, honeydew	spring	Cellulose Park	dark	A033L# F10.A0F2Y Honey, polyfloral, QUALITATIVE INFO = Dark
HDW-20SU	Multifloral, honeydew	summer	Cellulose Park	dark	A033L# F10.A0F2Y Honey, polyfloral, QUALITATIVE INFO = Dark

**Table 5:** Macro- and micro-elements and heavy metals (mg/Kg).

Honey Sample	Al	B	Ca	Cr	Cu	Fe	K	Mg	Mn	Na	Ni	P	Pb	Zn
AIL-19SP	0.50	3.6	89.6	0.091	0.20	0.23	765.0	36.4	0.30	38.4	0.004	57.2	0.02	0.00
TIL-19SP	0.08	1.8	112.4	0.034	0.11	0.62	1642.3	76.4	0.47	12.6	0.003	36.1	0.02	0.14
BLO-19SP	0.00	2.3	56.2	0.013	0.12	0.14	696.1	22.4	0.38	19.3	0.000	50.2	0.00	0.30
HDW-19SU	0.15	2.7	100.8	0.031	0.20	0.56	1412.7	121.2	0.99	52.9	0.000	85.7	0.00	0.18
HDW-20SP	0.00	3.4	53.1	0.005	0.47	0.00	3001.4	162.6	1.51	137.8	0.113	144.7	0.01	0.00
CAS-19SU	0.00	1.9	89.1	0.023	0.24	0.00	4079.2	61.0	0.64	0.4	0.000	45.4	0.11	0.00
HDW-21SP	0.00	3.3	45.6	0.009	0.28	0.00	3998.6	175.8	1.35	196.8	0.020	189.1	0.00	0.00
HDW-20SU	0.00	3.3	142.8	0.052	0.43	0.00	2645.6	192.3	1.35	85.9	0.041	162.8	0.13	0.00

## Data Availability

The authors declare that the data supporting the findings of this study are available within the paper. All the other data are available on reasonable request from the authors.
